# Rates of influenza vaccination in older adults and factors associated with vaccine use: A secondary analysis of the Canadian Study of Health and Aging

**DOI:** 10.1186/1471-2458-4-36

**Published:** 2004-08-11

**Authors:** Melissa K Andrew, Shelly McNeil, Heather Merry, Kenneth Rockwood

**Affiliations:** 1 Division of Geriatric Medicine, Department of Medicine, Dalhousie University, 1421-5955 Veterans Memorial Lane, Halifax, Nova Scotia, Canada B3H 1C6; 2 Department of Public Health Policy, London School of Hygiene and Tropical Medicine, London, UK; 3 Division of Infectious Disease, Department of Medicine, Dalhousie University, Halifax, Canada; 4 Clinical Trials Research Centre, Dalhousie University, Halifax, Canada

## Abstract

**Background:**

Influenza vaccination has been shown to reduce morbidity and mortality in the older adult population. In Canada, vaccination rates remain suboptimal. We identified factors predictive of influenza vaccination, in order to determine which segments of the older adult population might be targeted to increase coverage in influenza vaccination programs.

**Methods:**

The Canadian Study of Health and Aging (CSHA) is a population-based national cohort study of 10263 older adults (≥ 65) conducted in 1991. We used data from the 5007 community-dwelling participants in the CSHA without dementia for whom self-reported influenza vaccination status is known.

**Results:**

Of 5007 respondents, 2763 (55.2%) reported having received an influenza vaccination within the previous 2 years. The largest predictive factors for flu vaccination included: being married (57.4 vs. 52.6%, p = 0.0007), having attained a higher education (11.0 vs. 10.3 years, p < 0.0001), smoking (57.1% vs. 52.9%, p = 0.0032), more alcohol use (57.9% of those who drank more vs. 53.2% of those who drank less, p = 0.001), poorer self-rated health (54.1% of those with good self-rated health vs. 60.6% of those with poor self-rated health, p = 0.0006), regular exercise (56.8% vs. 52.0%, p = 0.001), and urban living (55.8% vs. 51.0%, p = 0.03).

While many other differences were statistically significant, most were small (e.g. mean age 75.1 vs. 74.6 years for immunized vs. unimmunized older adults, p = 0.006, higher Modified Mini Mental Status Examination score (89.9 vs. 89.1, p < 0.0001), higher comorbidity (2.7 vs. 2.3 comorbidities, p < 0.0001).

Residents of Ontario were more likely (64.6%) to report vaccination (p < 0.0001), while those living in Quebec were less likely to do so (48.2%, p < 0.0001). Factors retaining significance in a multivariate analysis included older age, higher education, married status, drinking alcohol, smoking, engaging in regular exercise, and having higher comorbidity.

**Conclusions:**

The vaccination rate in this sample, in whom influenza vaccination is indicated, was low (55.2%). Even in a publicly administered health care setting, influenza vaccination did not reach an important proportion of the elderly population. Whether these differences reflect patient preference or access remains to be determined.

## Background

Influenza has the potential to cause serious complications (chiefly viral and bacterial pneumonia, with attendant mortality and morbidity), especially in the older adult (>= 65) population and in those with comorbid illness [1-3]. Influenza vaccination is demonstratively safe, effective [4-6], and cost-effective [7,8], and current guidelines in both Canada and the United States recommend that the vaccine be administered yearly to all individuals over the age of 65 and to all residents of Long Term Care Facilities [9,10].

Despite current evidence and recommendations, influenza vaccination rates remain suboptimal. Estimates of vaccine coverage in high-risk groups (including older adults) range from 10–40% in the UK [11], and 45–68% in the United States [8,12,13]. A 1993 Canadian study found that 57.5% of community-dwelling Albertans over the age of 65 had been vaccinated within the past 12 months [14]. A question about influenza vaccination included in the 1991 Statistics Canada General Social Survey found that 44.8% of randomly sampled community dwelling individuals aged 65 and older had been vaccinated during the 1990/91 flu season [15]. A Canadian study of vaccination rates among residents of long term care (LTC) facilities reported 79% coverage for the 1990/91 season, increasing to 83% in 1998/99 [16].

To our knowledge, no previous studies have examined predictors of vaccination in community-dwelling older Canadians. The objectives of our study were to determine the vaccination rate in this community-dwelling population of older Canadians and to identify factors predictive of influenza vaccination, in order to determine which segments of the older adult population should be targeted to achieve better coverage in influenza vaccination programs.

## Methods

### Sample/study population

**Figure 1:**
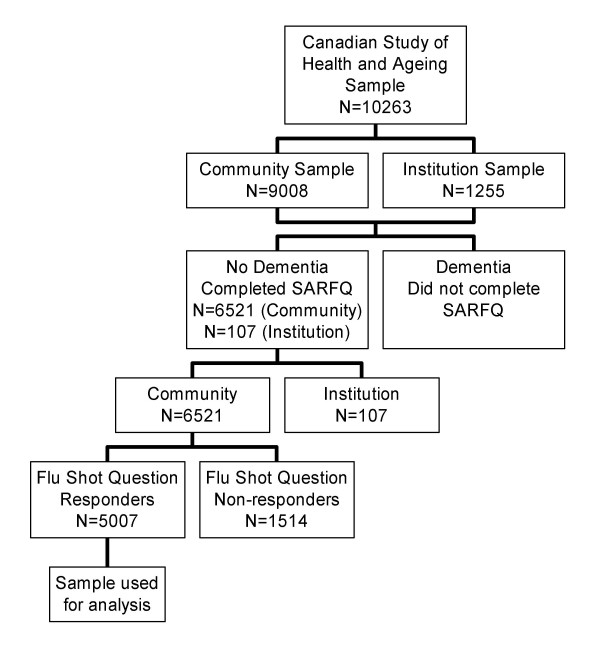
Derivation of the study population.

The Canadian Study of Health and Aging (CSHA) is a population-based representative sample drawn from Canadians over the age of 65, designed to study the prevalence, incidence, and risk factors for development of dementia [17]. A technical report providing details on sampling, design and measurement is available elsewhere [18]. Briefly, in 1991 and 1992, data were collected from 10,263 older adults: 9008 community-dwelling and 1255 in Long Term Care Facilities (LTCF). Those in the community were randomly selected from medicare lists (or the Enumeration Composite Record in Ontario), and institutionalized individuals were randomly selected from stratified random samples of institutions in each region. Individuals excluded were residents of the Yukon and Northwest Territories, those living on Aboriginal reserves or military bases, or those with life-threatening illnesses (examples cited include terminal cancer or conditions requiring life support). A Self-Administered Risk Factor Questionnaire (SARFQ) was completed by the non-demented community-dwelling participants in the study. The SARFQ included demographic questions and addressed issues of lifestyle, medical and family history, and medication use. From these questions a fitness/frailty scale was derived, that recognizes seven levels, from most fit (= 1) to most frail (= 7) [19]. A question on immunization history was asked, in which respondents were asked whether they had received the influenza vaccination, and if yes, approximately how many times they had had received it and the year of their most recent immunization. The sample population used in this study includes all participants who completed the SARFQ for whom influenza vaccination status could be determined based upon their answers. The derivation of the study population is shown in Figure 1.

### Statistical analysis

Respondents were designated vaccinated if they reported having had an influenza vaccine within the 2 years preceding completion of the SARFQ. As the data were collected over a two-year period, whereas the question about the last vaccination asked for a date, we designated as unvaccinated those who did not report receiving a vaccination within the previous 2 years. Vaccinated and unvaccinated respondents were compared; as a first step crude analyses were done taking each variable individually (not adjusted for any others). Chi-squared test or Fisher's exact test was used for categorical variables and one-way ANOVA was used for continuous variables. Characteristics analyzed included age, gender, education, region of residence, marital status, smoking status, alcohol intake, self-assessed health status, exercise, diagnosis of dementia, Modified Mini-Mental State Examination (3MS)[20] score, number of comorbidities, and urban *vs*. rural residence. As a second step, a multivariate analysis done by stepwise selection of parameters found to be significant in the univariate analysis was then conducted. Analyses comparing responders *vs*. non-responders to the relevant SARFQ questions as well as comparing respondents who reported first-time *vs*. regular influenza vaccination were also undertaken. The possibility of interaction between variables was considered. We included in the multivariable model interaction terms of pairs of variables where interaction was plausible (between smoking and drinking, age and frailty according to the frail scale, and region of residence and urban/rural dwelling).

## Results

Influenza vaccination status could be determined for 5007 (76.8%) of the 6521 non-demented, community-dwelling participants in the CSHA who completed the SARFQ. Of these, 2763 (55.2%) reported having received at least one influenza vaccination within the previous two years.

The univariate analysis, in which each variable was examined individually without adjusting for the effects of others, showed several differences between the demographics, region of residence, lifestyle, and health status of the two groups (Table 1). Immunized older adults were on average more highly educated, and being married (living with a partner or spouse) was another significant predictor of vaccination. Urban dwellers in general, and residents of Ontario were significantly more likely to have been immunized than those living elsewhere in Canada. The lowest self-report vaccination rates were found in Quebec.

**Table 1:** Univariate analysis of vaccinated vs. unvaccinated community dwelling older Canadians

**Risk Factor**	**Vaccinated (n = 2763)**	**Unvaccinated (n = 2244)**	**OR (95% C.I.)**	**P value**
Age mean (SD)	75.1 (6.5)	74.6 (6.7)	1.01 (1.00–1.02)	0.0056
Sex				
Male (%)	1162 (56.8)	883(43.2)	1.00	0.0527
Female (%)	1601 (54.1)	1361(45.9)	0.89 (0.80–1.00)	
Education mean years (SD)	11.0 (3.7)	10.3 (3.7)	1.05 (1.04–1.07)	<0.0001
Region				
Atlantic (%)	471 (54.1)	399 (45.9)	1.00	
Quebec (%)	453 (48.2)	486 (51.8)	0.79 (0.66–0.95)	<0.0001
Ontario (%)	568 (64.6)	311 (35.4)	1.55 (1.28–1.88)	
Prairies (%)	540 (55.6)	431 (44.4)	1.06 (0.88–1.28)	
BC (%)	731 (54.2)	617 (45.8)	1.00 (0.85–1.19)	
Current Marital Status				
Not married (%)	1197 (52.6)	1078 (47.4)	1.00	0.0007
Married (%)	1566 (57.4)	1163 (42.6)	1.21 (1.08–1.36)	
Smoker				
No (%)	1232 (52.9)	1095 (47.1)	1.00	0.0032
Yes (%)	1495 (57.1)	1122 (42.9)	1.18 (1.06–1.32)	
Alcohol				
No (%)	1590 (53.2)	1399 (46.8)	1.00	0.0012
Yes (%)	1133 (57.9)	824 (42.1)	1.21 (1.08–1.36)	
Self-Rated Health				
Not Good/Very Poor (%)	502 (60.6)	326 (39.4)	1.00	0.0006
Very/Pretty Good (%)	2254 (54.1)	1912 (45.9)	0.77 (0.66–0.89)	
Regular Exercise				
No (%)	893 (52.0)	824 (48.0)	1.00	0.0014
Yes(%)	1815 (56.8)	1382 (43.2)	1.22 (1.08–1.36)	
3MSE mean score (sd)	89.9 (6.4)	89.1 (6.4)	1.02 (1.01–1.03)	<0.0001
No. of comorbidities mean (sd)	2.7 (1.7)	2.3 (1.6)	1.15 (1.11–1.20)	<0.0001
Geography				
Urban (%)	2442 (55.8)	1934 (44.2)	1.00	0.0274
Rural (%)	320 (51.1)	306 (48.9)	0.83 (0.70–0.98)	

Of note, smokers were significantly more likely than non-smokers to have received the influenza vaccination. The same was true of individuals who consumed alcohol regularly when compared with those who drank rarely or not at all. Regular exercise was found to be a predictive factor for vaccination. Those who had received the vaccination had significantly more health problems, as did those who saw themselves as being in poorer health.

In the multivariate analysis (Table 2) predictive factors for immunization that retained significance after adjusting for the effects of other variables include older age, higher level of education, being married, smoking, engaging in regular exercise, and having more co-morbid illnesses. Interaction terms describing interaction between age and frailty, smoking and drinking, and region of residence and urban/rural dwelling did not retain significance in the multivariable model, suggesting that there was no statistically significant interaction between these pairs of variables.

**Table 2:** Multivariate analysis comparing vaccinated and unvaccinated community-dwelling older Canadians

**Risk Factor**	**Odds Ratio**	**95% C.I.**	**p value**
Older Age	1.02	1.01–1.06	<0.0001
More Education	1.05	1.03–1.07	<0.0001
Currently Married	1.29	1.14–1.47	<0.0001
Smoking	1.14	1.01–1.30	0.0401
Drinking	1.51	1.01–1.31	0.0358
Regular Exercise	1.25	1.10–1.42	0.0004
Region			
Atlantic	1.00	---	
Quebec	0.94	0.77–1.14	<0.0001
Ontario	1.48	1.21–1.82	
Prairies	0.99	0.82–1.20	
BC	0.80	0.67–0.97	
More Comorbidity	1.18	1.140–1.226	<0.0001

To investigate the impact of response bias, we compared those who had answered the influenza vaccination question and the 23.2% of the original sample who had not (Table 3). Non-responders were more likely to be rural residents and to drink alcohol regularly. Age, 3MS score, self-rated health, and number of comorbidities also differed between the groups: non-responders were more likely to be younger, healthier, and to have better self-rated health and higher 3MS scores. Several of the same characteristics (age, number of comorbidities, self-rated health, region of residence and urban vs. rural living) also differed between regular and first time vaccination users (Table 4). Repeat users of the influenza vaccine (i.e. those who reported at least two past vaccinations) were slightly older (75.3 *vs*. 74.1 years, p < 0.0001), had more comorbid illness (2.7 *vs*. 2.5, p = 0.0011), and poorer self-rated health (81.7% poor health *vs*. 77.9% good health, p = 0.045). Residents of Atlantic Canada (75.1% *vs*. 79.3% residing elsewhere, p = 0.0003) and those living in rural areas (79.3% of urban *vs*. 73.8% of rural-dwellers, p = 0.12) were less likely to be regular users.

**Table 3:** Analysis of responders vs. non-responders to questions relating to influenza vaccination in the SARFQ

**Characteristic**	**Value Responders (n = 5007)**	**Value Non-responders (n = 1514)**	**p value**
Age mean (SD)	74.9	73.4	<0.0001
Sex			
Male (%)	76.9	23.1	0.8416
Female (%)	76.7	23.3	
Education mean (SD)	10.7	10.7	0.8164
Region			
Atlantic (%)	71.6	28.4	
Quebec (%)	78.9	21.1	<0.0001
Ontario (%)	69.4	30.6	
Prairies (%)	70.1	29.9	
BC (%)	92.1	7.9	
Current Marital Status			
Not married (%)	77.1	22.9	0.5742
Married (%)	76.5	23.5	
Smoker			
No (%)	78.0	22.0	0.1888
Yes (%)	76.6	23.4	
Alcohol			
No (%)	82.4	17.6	<0.0001
Yes (%)	75.8	24.2	
Self-Rated Health			
Not Good/Very Poor (%)	82.4	17.6	<0.0001
Very/Pretty Good (%)	75.8	24.2	
Regular Exercise			
No (%)	77.8	22.2	0.6263
Yes(%)	77.3	22.7	
MMMSE mean (sd)	89.6	90.1	0.0071
No. of comorbidities mean (sd)	2.5	2.1	<0.0001
Geography			
Urban (%)	77.7	22.3	<0.0001
Rural (%)	70.7	29.3	

SARFQ = Self-Administered Risk Factor Questionnaire in the Canadian Study of Health and Aging

**Table 4:** Characteristics of regular vs. first time influenza vaccine users

**Risk Factor**	**Regularly Vaccinated**	**First Time Vaccinated**	**P value**
Age mean years (SD)	75.3 (6.7)	74.1 (6.7)	<0.0001
Sex			
Male (%)	79.5	20.5	0.29
Female (%)	78.9	22.0	
Education mean years (SD)	11.0 (3.8)	10.8 (3.8)	0.27
Region			
Atlantic (%)	75.1	24.9	0.0003
Quebec (%)	83.7	16.3	
Ontario (%)	81.5	18.5	
Prairies (%)	78.2	21.8	
BC (%)	75.5	24.5	
Current Marital Status			
Not married (%)	79.2	20.8	0.45
Married (%)	78.1	21.9	
Smoker			
No (%)	78.3	21.7	0.73
Yes (%)	78.8	21.2	
Alcohol			
No (%)	78.4	21.6	0.83
Yes (%)	78.7	21.3	
Self-Rated Health			
Not Good/Very Poor (%)	81.7	18.3	0.045
Very/Pretty Good (%)	77.9	22.1	
Regular Exercise			
No (%)	80.2	19.8	0.086
Yes(%)	77.5	22.5	
3MSE mean score (sd)	89.9 (6.4)	89.7 (6.4)	0.48
No. of comorbidities mean (sd)	2.7 (1.7)	2.5 (1.7)	0.0011
Geography			
Urban (%)	79.3	20.7	0.012
Rural (%)	73.8	26.2	

## Discussion

Our data were collected in the early 1990s and may therefore not reflect current practice patterns. Although the Canadian Study of Health and Aging was not designed to study influenza vaccination, it does provide a large and unique data set of primarily community-dwelling older Canadians, and is therefore potentially useful in the examination of health-related risk factors and demographics that may influence decisions to vaccinate. Given the importance of influenza vaccination in the prevention of significant morbidity and mortality in populations at risk, the vaccination rate of 55.2% in our community-dwelling sample of older adults is concerning. People who were not vaccinated tended to be younger, non-smokers and to have fewer co-morbid illnesses. They were also found to have a lower level of education, not to be married and not to engage in regular exercise. These were the factors that retained statistical significance in the multivariable analysis, suggesting that these associations are unlikely to have arisen due to confounding by any of the variables investigated.

Vaccination has previously been studied in the CSHA, but only in relation to its status as a potentially protective factor with respect to cognitive impairment [21]. The weight of evidence derived from re-examination of large databases is less than that derived from specifically designed trials, however this method still maintains an important role in epidemiological research. For example, evaluation of systematic problems can be used to help develop targeted efforts in improving vaccination rates. Our data do not include information on institutionalized older adults, where it might be expected that at-risk profiles would vary, and where vaccination rates are generally higher [16].

Another important source of potential error is our reliance on self-reported immunization status. However, self-report of influenza vaccination status in elderly outpatients has been found to be highly sensitive and moderately specific when checked against medical record documentation [22].

Other studies have identified predictive factors for vaccination in other countries [8,12]. An American study found that patients with more health conditions, higher rates of use of health care resources, and history of pneumonia were more likely to be vaccinated, while non-vaccinated individuals were older and more likely to have dementia or stroke [8]. An Iowa study identified a number of factors associated with the receipt of both influenza and pneumococcal vaccines: age over 70, self-owned residence, working, increased number of medical conditions, current prescription medication, and a physician visit within the past year. Geography (rural vs. urban living) was unrelated to vaccine receipt [12].

Our data suggest that there appears to be an important degree of targeting of vaccination resources within the older adult population to people who are less healthy. Even within these higher risk groups, however, immunization is incomplete. Perhaps such people are perceived by their health care providers as being at higher risk from influenza infection, and are thus more likely to be immunized. Similar explanations may account for the higher vaccination rates among smokers and those who consumed more alcohol. However, according to the current Canadian guidelines [9], influenza vaccination is indicated for all persons 65 years of age and over. If it is the case that those at the younger end of this age group are less likely to be vaccinated, as our study suggests, more must be done to ensure that vaccination is reaching its entire target population.

Regular exercise was shown to be a factor predictive of influenza vaccination. Interestingly, this association may have been confounded by generally health-protective behaviour (which might be expected to be associated with both regular exercise, the explanatory variable, and vaccination, the outcome), given that individuals exhibiting healthy lifestyle choices (such as exercise) may have been more likely to have sought preventative health care and to have visited their health care providers more regularly, thus providing more opportunity to be vaccinated. Regular exercise did, however, retain significance in our multivariate analysis.

The regional differences identified in our study may point to geographic differences in access to influenza vaccination, although the general milieu and level of awareness in both the medical community and society at large may also be significant. These regional differences suggest that certain areas may benefit from targeting of vaccination efforts. However, over and above questions of health policy and public health education, the question of access to vaccination is of vital importance if we are to achieve acceptable rates of coverage in this target population. The finding that rural residence is a negative predictor of vaccination is particularly concerning, and points to the larger equity issue of how uptake of influenza vaccination can be improved outside of major urban centers. However, our finding that rural *vs. *urban residence did not retain statistical significance in the multivariable regression model suggests that this crude association may be due to confounding by other factors.

Our analysis comparing non-responders with those for whom influenza vaccination status was known on the basis of the SARFQ showed that response bias was indeed likely. Non-responders were more likely to drink more alcohol, a factor which was associated with being immunized. However, several factors which were associated with not being immunized, including younger age, good self-rated health, having fewer comorbidities, and residence in a rural area, were more prevalent among non-responders. This over-representation of a number of factors predictive of non-immunization among non-responders suggests that our results may well have been influenced by response bias. For example, we may have over-estimated the prevalence of vaccination in this population.

The analysis comparing regular users of the influenza vaccine with those who reported first-time immunization in the SARFQ demonstrated a number of factors that differed between the two groups. The finding that younger age is associated with first time vaccination may be at least partially explained by those at the younger end of the > = 65 age group having spent less time in this "target group" for vaccination. As such, they would be more likely to be receiving the vaccination for the first time. The effect of age may also play a role in the finding that those with better self-assessed health and fewer comorbidities were more likely to be first-time users (as they may also have been younger). However, being in better health was also a predictive factor for non-vaccination within the previous two years, as was rural residence, suggesting that these factors may influence decisions to initiate as well as to sustain influenza vaccination over subsequent years.

## Conclusions

Despite current recommendations and proven benefit, influenza vaccination rates in our Canadian sample were suboptimal; only 55.2% of older adults in our representative sample reported being vaccinated within the past two years. The predictive factors identified in this analysis may facilitate the study of targeting of older adults in future vaccination programs.

## Competing interests

SM and HM have been involved in trials of Influenza vaccines funded by ID Biomedical. Neither has received any personal financial compensation for their involvement. MKA and KR have no competing interests.

## Author contributions

MKA wrote the first draft, and reviewed each of the analyses. SM & KR reviewed the analyses and revised the manuscript. KR is a co-Principal Investigator of the CSHA. HM did the statistical analyses. All authors read and approved the final manuscript.

## Pre-publication history

The pre-publication history for this paper can be accessed here:


